# Achieving Sustained Hair Repigmentation in Poliosis Circumscripta: Retreatment Outcomes Using Exosomes and Fractional Picosecond Laser

**DOI:** 10.1111/jocd.70115

**Published:** 2025-03-15

**Authors:** Suparuj Lueangarun, Byong Seung Cho, Therdpong Tempark

**Affiliations:** ^1^ Department of Aesthetic Medicine, College of Integrative Medicine Dhurakij Pundit University Bangkok Thailand; ^2^ Division of Dermatology DeMed Clinic Center Bangkok Thailand; ^3^ ExoCoBio Exosome Institute (EEI) ExoCoBio Inc. Seoul Republic of Korea; ^4^ Department of Pediatrics, Faculty of Medicine King Chulalongkorn Memorial Hospital, Chulalongkorn University Bangkok Thailand

**Keywords:** androgenetic alopecia (AGA), exosomes, fractional picosecond laser (FPL), hair repigmentation, poliosis circumscripta, rose stem cell exosomes

Dear Editor,

I would like to respond to the published article titled “Hair Repigmentation of Poliosis Circumscripta in an Androgenetic Alopecia (AGA) Patient Treated with Exosomes and Fractional Picosecond Laser (FPL)” [1] and provide additional insights regarding the treatment and mechanisms of action discussed.

Our study further explores the promising outcomes observed with topical exosome and FPL therapy for the treatment of poliosis circumscripta. After completing four treatments, the patient returned for follow‐up 10 months after the initial sessions. At that time, the patient reported continued hair thickening but noted a slight increase in the area of white hair compared to the last follow‐up, suggesting partial regression of the repigmented areas (Figure 1).

**FIGURE 1:**
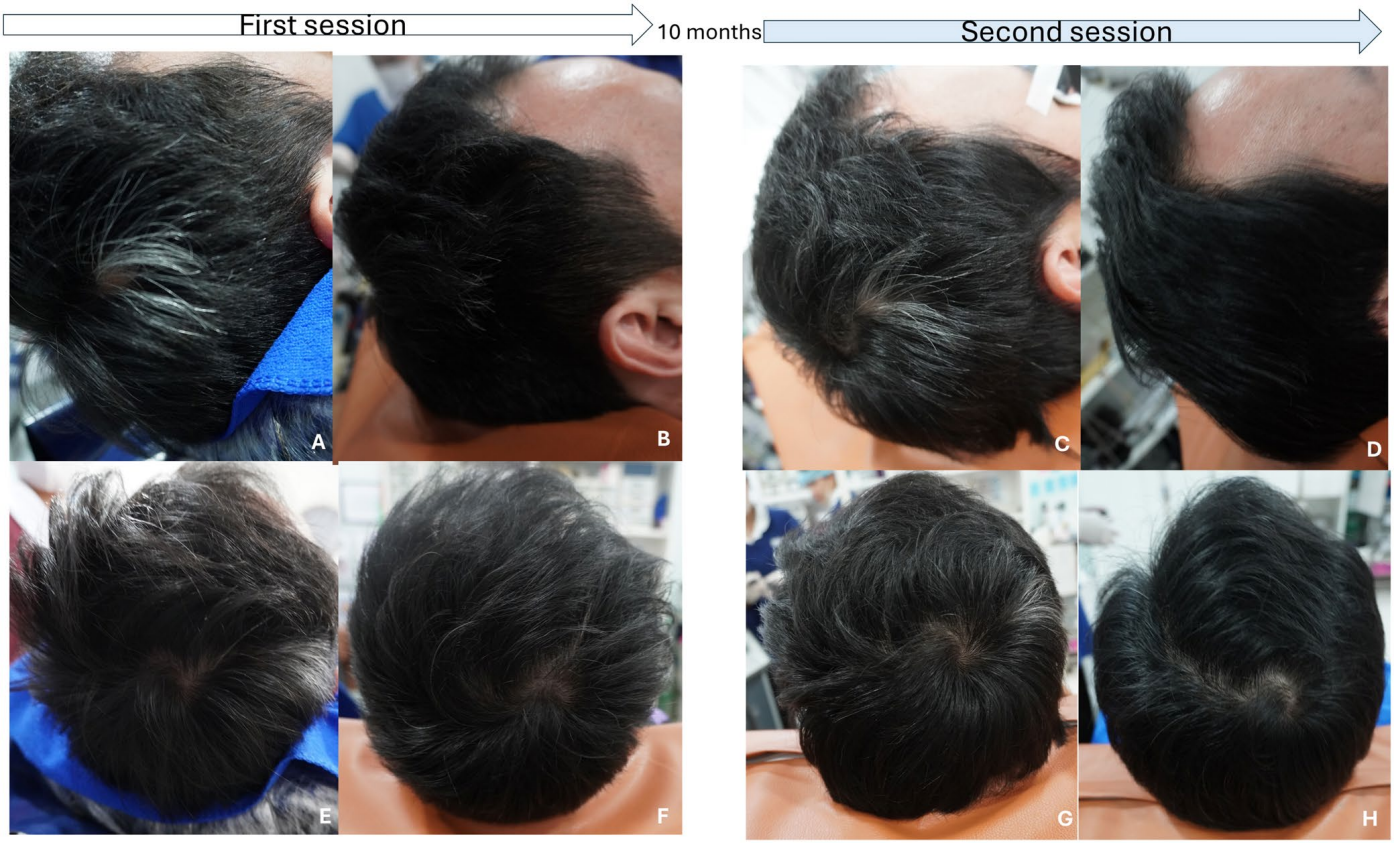
Hair regrowth and repigmentation after FPL and RSCEs therapy. The images illustrate the progression of hair restoration and repigmentation. The upper row represents the right lateral view, while the lower row represents the vertex view: Before treatment (A and E), 4 weeks after the 4th treatment of the first session (B and F), at a 10‐month follow‐up after the first treatment session (C and G), and 4 weeks after 3 sessions of the second treatment session (D and H).

Subsequently, the patient underwent three additional treatment sessions following the same protocol, which included topical rose stem cell exosomes (RSCEs, ASCE+ HRLV; ExoCoBio Inc., Seoul, Korea), combined with FPL as previously described [1]. Currently, most exosome‐based products are classified as cosmetic products. However, in Thailand, certain exosome formulations, such as ASCE+ (ExoCoBio Inc., Seoul, Korea), have been registered as medical devices. The same promising results were observed, as evaluated by both clinical and dermoscopic examinations. The patient's scalp showed significant hair regrowth and repigmentation of the poliosis patch, with an increase in black hair density.

At the 10‐month follow‐up after the first treatment session, the results showed significant improvement compared to previous sessions. Hair regrowth was maintained, and repigmentation of the poliosis patch continued to progress. Dermoscopic evaluation (DermLite DL5, San Juan Capistrano, CA, USA) revealed an increase in black hair density and a decrease in white hair density, with the poliosis area showing more black hairs than before (Figure 2).

**FIGURE 2:**
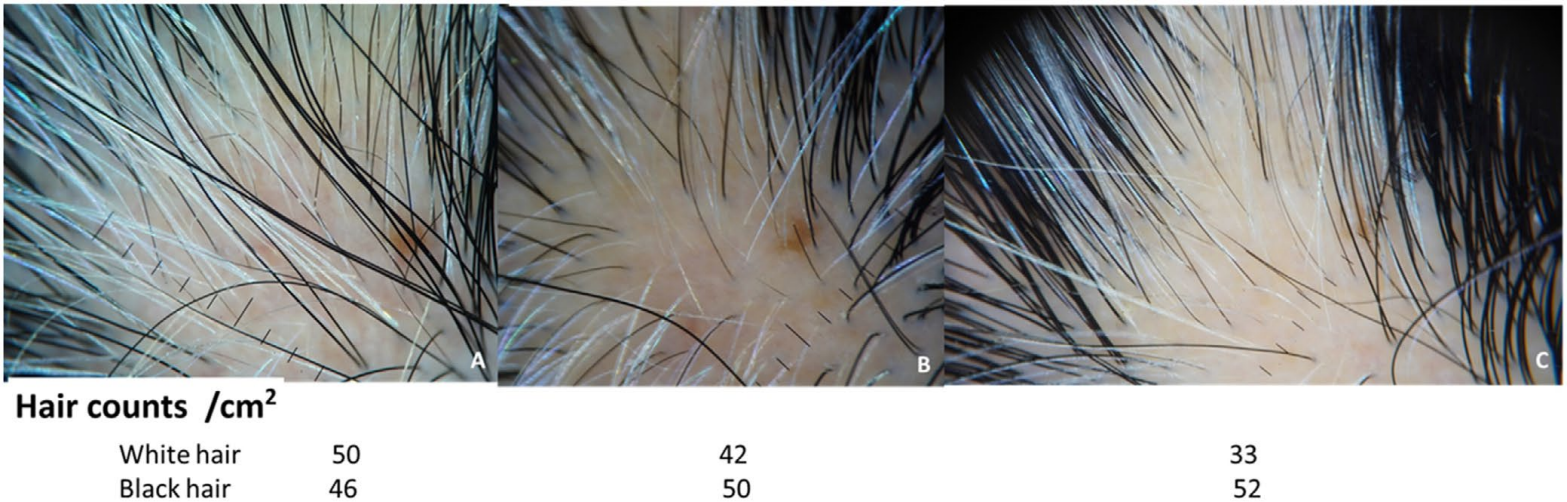
Dermoscopic examination with black and white hair counts (per cm^2^) at baseline (A), 10 months after the first treatment session (B), and 4 weeks after the second treatment session (C). Following the second treatment period, the results show a reduction in the area of white hair and an increase in black hair density within the poliosis patch, with the nevus serving as a consistent reference point.

Additionally, regrowing black hair was observed in the poliosis area, with the proximal parts of the hair shafts showing black pigmentation, while the distal portions remained white (Figure 3).

**FIGURE 3:**
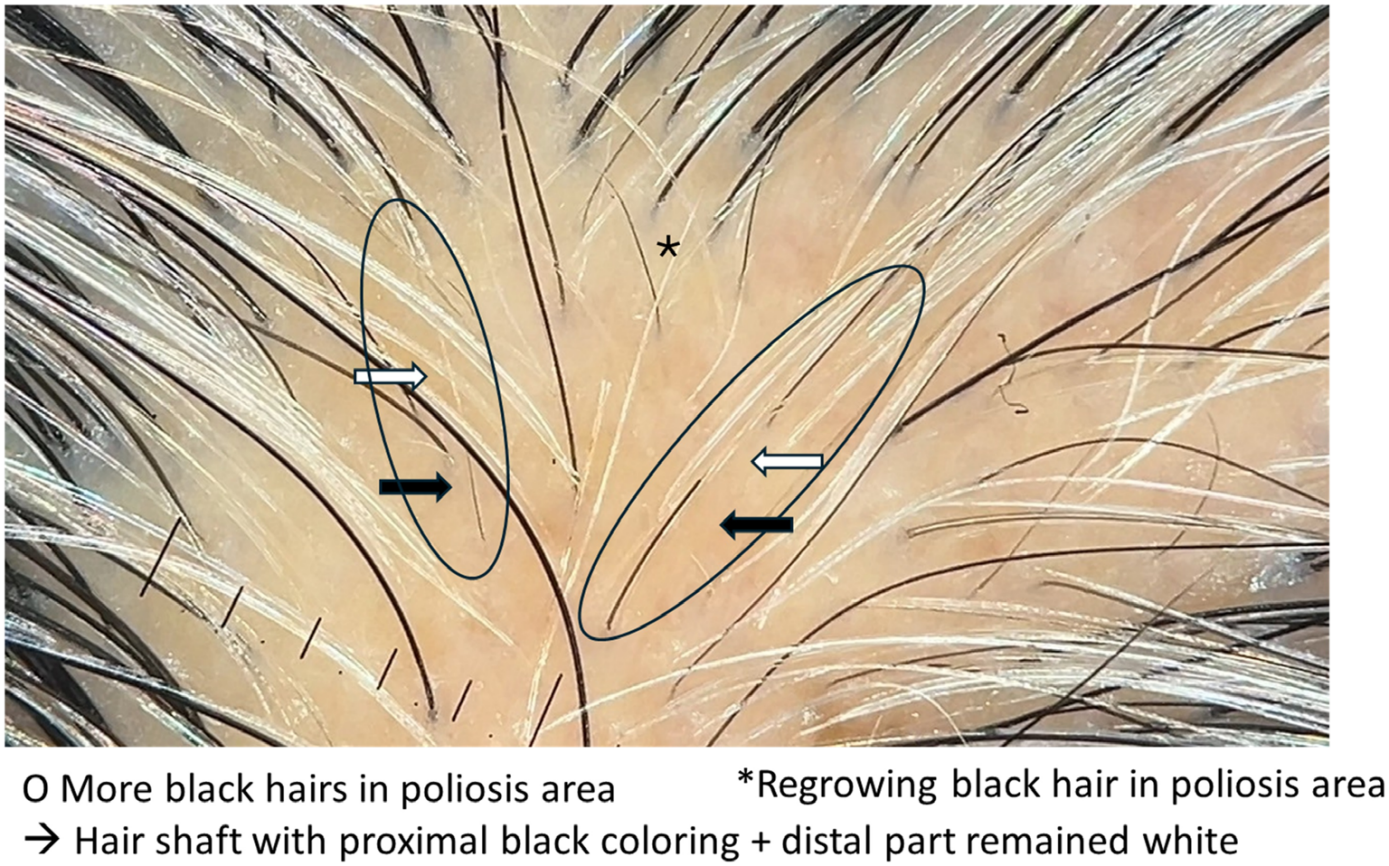
Illustration of hair pigmentation recovery in poliosis‐affected areas. The proximal segments of the hair shafts regain black pigmentation, while the distal parts remain white. Black hair regrowth is also observed in the poliosis‐affected area.

1

The combination of FPL and RSCEs therapy demonstrated promising results in addressing both hair loss and the repigmentation of poliosis circumscripta and AGA in this patient, confirmed through two treatment sessions. While the initial response showed hair thickening and repigmentation, the patient's condition following retreatment remained encouraging. The retreatment, which involved three additional sessions of exosomes and FPL therapy, yielded similar results in terms of repigmentation, reaffirming the reproducibility of this therapeutic approach.

Our findings further highlight the role of exosomes in stimulating melanocyte proliferation and melanin synthesis. Studies identified specific microRNAs (miRNAs) within exosomes, such as miR‐21a‐5p, miR‐200c, and miR‐3196, which appear to enhance melanogenesis and promote melanin production in melanocytes [2]. We postulate that the repigmentation effect resulting from miR‐200c, detected in RSCEs [3], positively regulates melanogenesis by targeting SOX1. This leads to the upregulation of MITF‐dependent genes involved in melanogenesis [4], ultimately resulting in hair repigmentation in poliosis circumscripta.

However, this case report acknowledges limitations, including the potential role of spontaneous repigmentation and confounding from concurrent FPL therapy, while highlighting promising results for the combined approach in treating poliosis and promoting hair repigmentation. From the review of the literature, reports of hair repigmentation have been associated with medications and certain procedures, but not with laser treatments. Hair repigmentation has been observed with medications such as monoclonal antibodies, tyrosine kinase inhibitors, and immunomodulatory drugs, likely through immune‐modulating or cytokine pathways [5, 6]. Procedure‐induced repigmentation, as seen in micro‐injuries from Mohs surgery or phototherapy, activates hair follicle melanocyte stem cells via pathways such as Wnt/β‐Catenin and EDN3/EDNRB [7].

Ongoing research seeks to clarify the role of exosomes in hypopigmentary conditions and refine treatment protocols, contributing to a deeper understanding of their therapeutic potential in clinical practice.

## Ethics Statement

All procedures performed in studies involving human participants were in accordance with the ethical standards of the 1964 Helsinki Declaration and its later amendments or comparable ethical standards.

## Consent

Informed consent was obtained from the participants in the study.

## Conflicts of Interest

The authors declare no conflicts of interest.

## Data Availability

The data that support the findings of this study are available from the corresponding author upon reasonable request.
